# The King at Cambridge

**Published:** 1915-02-20

**Authors:** 


### The King at Cambridge.

### 1st EASTERN GENERAL HOSPITAL VISITED.

Cambridge was for the second time in less than twelve months honoured by the King's presence on Thursday last week. His Majesty's last visit was in April 1914, to open the new wing of the Leys School. Being purely of a military character on this occasion, there was no civic reception.

On the preceding day it had been rumoured that his Majesty would visit Cambridge for the purpose of inspecting the troops, but for reasons that are obvious no statement was publicly made, and many, remembering the disappointment caused by rumours in the past, doubted the reliability of the present rumour, with the result that those who assembled in the line of route and on Parker's Piece were much smaller in number than would have been the case if a public announcement had been made.

The King, who arrived by special train, was accompanied by General Sir Ian Hamilton, the Commander-in-Chief of the Home Forces, General Bruce Hamilton, and Major Clive Wigram (Equerry). He was received by the General Officer Commanding the troops and other officers. He thereupon proceeded by motor-car to Parker's Piece.

As the King passed the Rest Home for Belgian Wounded Soldiers in Hills Road those of the Belgian wounded who were on the balcony cheered most heartily.

The parade took place on Parker's Piece, whose open space of twenty-two acres is such a feature of the centre of the town.

The troops, consisting of cavalry and infantry, were all on foot, including the General Officer Commanding and his Staff, there being present neither horses, cannon, nor wagons, as it would have been impossible to have arranged for the admission of such to the Piece.

At a position near the centre of the Piece as the bugle sounded a G it brought in reply myriad flashes of steel as the troops brought their rifles to the " Present " for the Royal salute, and the band played the National Anthem, during which the lines of steel were immovable and the spectators bared their heads.

His Majesty then inspected the troops in the following order: The cavalry, artillery, infantry, A.S.C., R.A.M.C., and the National Reserves. As his Majesty came near, each unit drew to attention.

With the Staff the King passed along the front of each unit, accompanied by the various commanders, pausing at intervals for a second. The pause at the National Reserve unit was of some minutes' duration, and his Majesty conversed for some time with Major Francis and Lieut. S. M. Jonas.

After the inspection, the King took his stand at the saluting base to witness the march past, which took nearly an hour.

Having inspected the signal section of the cavalry, the King motored to the 1st Eastern General Hospital, where he was received at the gate by the Commanding Officer, Colonel Griffiths, and other officers. The principal matron, Miss C. C. Crookenden (of Addenbrooke's), and the acting matron, Miss Newton, were presented.

The time which the King was able to spend at the hospital being limited, Colonel Griffiths conducted him to the far end of the hospital, where two of the wards were visited. The King spoke to many of those who had been wounded, expressing his sympathy with them.

The kitchen was visited, where the King saw the dinners which were ready for serving to the wards, and the methods of cooking were observed. Finally the King visited the operating theatre, where operations were actually in progress.

The King was deeply interested in the hospital, which is of the open-air type, and made many inquiries as to the arrangements for the patients under the late adverse conditions of weather.

Before leaving, permission was given for photographs to be taken of a group near the operating-theatre entrance. The Commanding Officer, the Registrar, the Quartermaster, the principal matron, Miss Crookenden, and the acting matron, Miss Newton, were included in the group.

The Royal procession, after leaving the hospital, passed to Hyde Park Corner, from which point his Majesty proceeded to the station, between the troops which lined the route two deep on either side, at walking pace. The station was reached a little before 1.20.

